# Pyomyositis of tensor fascia lata: a case report

**DOI:** 10.1186/1752-1947-2-236

**Published:** 2008-07-24

**Authors:** Korhan Ozkan, Koray Unay, Ender Ugutmen, Abdullah Eren, Engin Eceviz, Baransel Saygý

**Affiliations:** 1 Goztepe Research and Training Hospital, Department of Orthopedics and Traumatology, Istanbul, Turkey; 2 Fatih Sultan Mehmet Research and Training Hospital, Department of Orthopedics and Traumatology, Istanbul, Turkey

## Abstract

**Introduction:**

Pyomyositis is a disease in which an abscess is formed deep within large striated muscles.

**Case presentation:**

We report the case of a 10-year-old boy who presented with fever and a painful hip and was subsequently diagnosed with pyomyositis of the tensor fascia lata. In children with clinical and laboratory findings of inflammation in the vicinity of the hip joint, the differential diagnosis includes transient synovitis, an early stage of Legg-Calvé-Perthes disease, infectious arthritis of the hip, rheumatologic diseases and extracapsular infection such as osteomyelitis.

**Conclusion:**

To the best of the authors' knowledge, this is the first report of pyomyositis of the tensor fascia lata. Although pyomyositis is a rare disease and the differential diagnosis includes a variety of other commonly observed diseases, pyomyositis should be considered in cases where children present with fever, leukocytosis and localized pain.

## Introduction

Pyomyositis is a disease in which an abscess is formed deep within large striated muscles [1]. Outside the tropics, it is a rare disease [2]. Diagnosis is difficult due to the similarity of the symptoms with several infectious and inflammatory processes, mainly septic arthritis and transient synovitis. We describe the case of a 10-year-old boy admitted to the emergency unit with fever and a painful hip who was subsequently diagnosed with pyomyositis of the tensor fascia lata.

## Case presentation

A 10-year-old boy was admitted to the emergency unit with symptoms of fever and a painful hip. The child did not have any chronic disease or predisposing factors. He had a 20° left hip flexion contracture with a limited range of motion in rotations.

**Figure 1:**
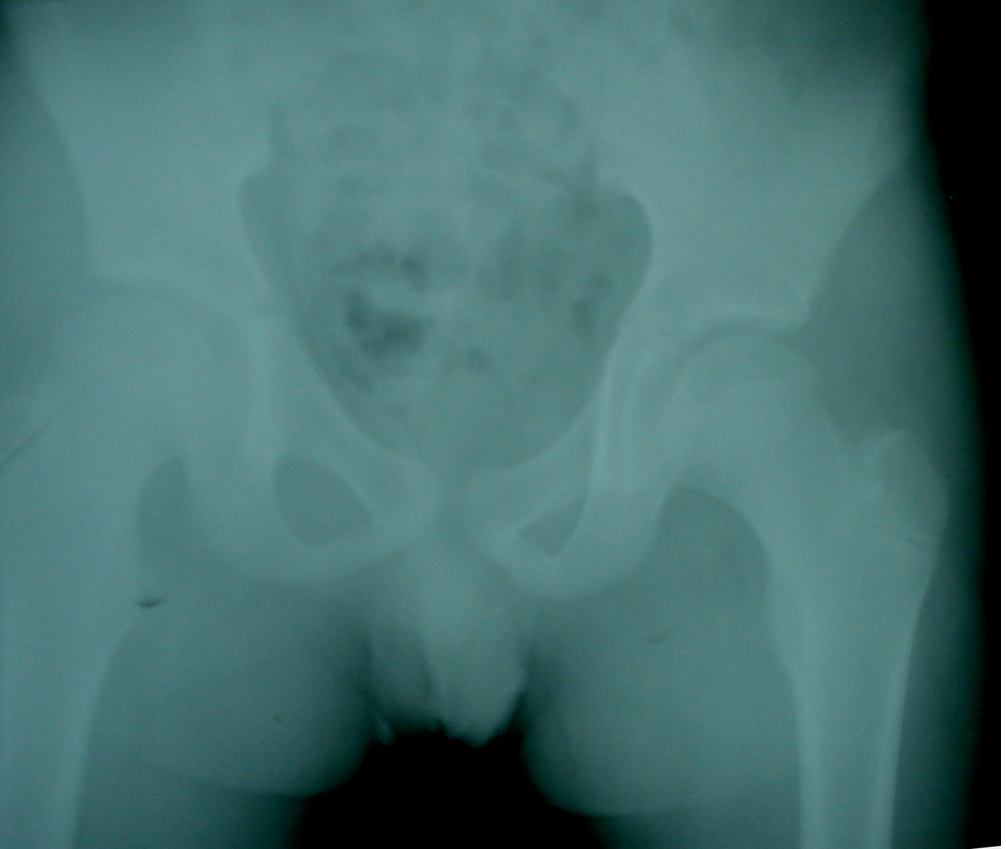
**A roentgenogram of the pelvis**. Anterior-posterior view.

**Figure 2:**
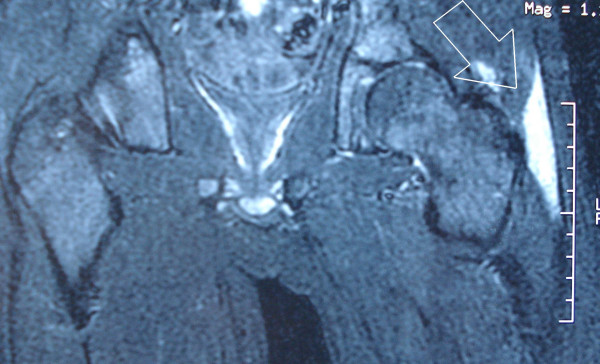
Magnetic resonance imaging of the pelvis showing fluid accumulation (arrow) at the tensor fascia lata.

Laboratory tests revealed a total leukocyte count of 14,100/mm^3 ^with 85% neutrophils. The erythrocyte sedimentation rate was 18 mm in the first hour, and the C-reactive protein concentration was within normal levels. Pharyngeal, urine and blood cultures and a chest X-ray were performed to investigate for other primary infections. Another origin of infection was not found. Since septic arthritis was suspected, needle aspiration of the hip was performed under fluoroscopy; it yielded no fluid. No abnormal view was seen in the pelvis roentgenogram (Figure 1). Magnetic resonance imaging (MRI) revealed fluid accumulation in the tensor fascia lata (Figure 2). Aspiration of this area yielded pus, and methicillin-sensitive *Staphylococcus aureus *was identified in the cultures. The final diagnosis was pyomyositis of the tensor fascia lata. The patient was administered antibiotic treatment (40 mg/kg/day cefazolin).

**Figure 3:**
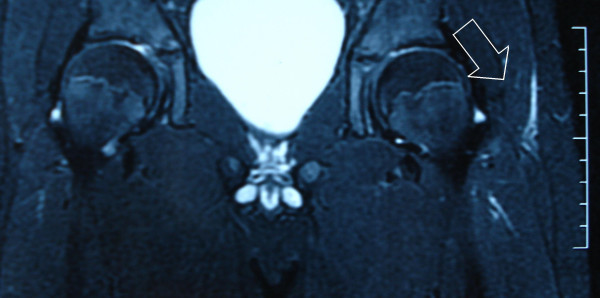
Control magnetic resonance imaging of the left hip (5 days after antibiotherapy) showing a significant decrease in the extent of involvement (arrow).

After 10 days of treatment, the leukocyte count returned to normal and the active and passive motion of the hip became completely painless. Control MRI of the left hip showed a significant decrease in the extent of involvement (Figure 3). The patient was discharged and cefazolin treatment continued for an additional 4 weeks.

## Discussion

We report pyomyositis of the tensor fascia lata in a 10-year-old boy presenting with fever and a painful hip. In children with clinical and laboratory findings of inflammation in the vicinity of the hip joint, the differential diagnosis includes transient synovitis, an early stage of Legg-Calvé-Perthes disease, infectious arthritis of the hip, rheumatologic diseases and extracapsular infections such as pyomyositis and osteomyelitis. There are also cases of pyomyositis presenting with septic pulmonary emboli, so a consultation with a pediatrician is required to eliminate septic pulmonary emboli [3]. Clinically, a high level of suspicion is required for the diagnosis of pyomyositis in patients presenting with fever, leukocytosis and localized pain [4], since this condition is extremely rare. To the best of the authors' knowledge, this is the first report of pyomyositis of the tensor fascia lata.

MRI is crucial for the accurate diagnosis of the location of infection and the extent of involvement. Since it provides a relatively higher rate of accuracy, MRI can prevent unnecessary surgery as a result of a misdiagnosis of septic arthritis [5,6]. Pyomyositis of the tensor fascia lata may simulate infectious arthritis of the hip, and awareness regarding this condition should facilitate earlier diagnosis and treatment.

The treatment for pyomyositis is the same as for other soft-tissue infections. Appropriate antibiotics are administered and surgical incision and drainage should be performed. Local heat application and immobilization are auxiliary treatment options. However, in our case, the clinical symptoms improved and the leukocyte count returned to normal levels with only antibiotherapy; hence, surgery was not required.

## Conclusion

We have reported the case of a young boy with pyomyositis of the tensor fascia lata. Although pyomyositis is a rare disease, rapid diagnosis with MRI is essential for these patients. Treatment is based on appropriate antibiotherapy. Surgery is indicated if the symptoms persist and if laboratory measures are unsuccessful in reducing the inflammation.

## Abbreviations

MRI: magnetic resonance imaging.

## Competing interests

The authors declare that they have no competing interests.

## Authors' contributions

KO and KU contributed to the conception and design, and carried out the literature research, manuscript preparation and manuscript review. EU and AE were involved in the literature review and helped draft part of the manuscript. EE contributed to the conception and design. BS supervised the writing and general management of the patient. KO, KU and AE revised the manuscript.

## Consent

Written informed consent was obtained from the patient's next-of-kin for publication of this case report and any accompanying images. A copy of the written consent is available for review by the Editor-in-Chief of this journal.
